# An injectable self-healing anesthetic glycolipid-based oleogel with antibiofilm and diabetic wound skin repair properties

**DOI:** 10.1038/s41598-020-73708-7

**Published:** 2020-10-22

**Authors:** Yadavali Siva Prasad, Sandeep Miryala, Krishnamoorthy Lalitha, Balasubramani Saritha, C. Uma Maheswari, Vellaisamy Sridharan, C. S. Srinandan, Subbiah Nagarajan

**Affiliations:** 1 Department of Chemistry, School of Chemical and Biotechnology, SASTRA Deemed University, Thanjavur, Tamil Nadu 613401 India; 2 Biofilm Biology Lab, Centre for Research in Infectious Diseases, School of Chemical and Biotechnology, SASTRA Deemed University, Thanjavur, Tamil Nadu 613401 India; 3 Department of Chemistry and Chemical Sciences, Central University of Jammu, Rahya-Suchani (Bagla), District-Samba, Jammu, J&K 181143 India; 4 Department of Chemistry, National Institute of Technology Warangal, Warangal, Telangana 506004 India

**Keywords:** Health care, Chemistry, Materials science, Nanoscience and technology, Antimicrobials, Bacteria, Biofilms, Molecular medicine

## Abstract

Globally, wound infections are considered as one of the major healthcare problems owing to the delayed healing process in diabetic patients and microbial contamination. Thus, the development of advanced materials for wound skin repair is of great research interest. Even though several biomaterials were identified as wound healing agents, gel-based scaffolds derived from either polymer or small molecules have displayed promising wound closure mechanism. Herein, for the first time, we report an injectable and self-healing self-assembled anesthetic oleogel derived from glycolipid, which exhibits antibiofilm and wound closure performance in diabetic rat. Glycolipid derived by the reaction of hydrophobic vinyl ester with α-chloralose in the presence of novozyme 435 undergoes spontaneous self-assembly in paraffin oil furnished an oleogel displaying self-healing behavior. In addition, we have prepared composite gel by encapsulating curcumin in the 3D fibrous network of oleogel. More interestingly, glycolipid in its native form demoed potential in disassembling methicillin-resistant *Staphylococcus aureus*, methicillin-susceptible *Staphylococcus aureus*, and *Pseudomonas aeruginosa* biofilms*.* Both oleogel and composite gel enhanced the wound skin repair in diabetic induced Wistar rats by promoting collagen synthesis, controlling free radical generation and further regulating tissue remodeling phases. Altogether, the reported supramolecular self-assembled anesthetic glycolipid could be potentially used for diabetic skin wound repair and to treat bacterial biofilm related infections.

## Introduction

An external injury to any of the skin or muscle tissues in the body is generally referred as wound, which ensue in a critical damage to membrane, extracellular matrices, blood vessels, cells, and organ structures. In order to prevent bacterial infections of wounds caused by both traumatic and surgical injury, special medical attention is required because of the involvement of various dynamic process involving coagulation, inflammation, proliferation, and tissue remodeling^1^. Generally, sutures, staples and adhesive tapes are common wound closure techniques adopted by the medical practitioners despite its drawbacks such as high rate of infection, inflammatory reaction, nerve damage, fluid leakage, granuloma and scar tissue formation. It is worth mentioning that the delayed closure of chronic wounds in diabetic patients encompasses improper angiogenesis, enormous production of free radicals, lack of cell to cell communication, suppressed cell migration, substandard production of extracellular matrix (ECM) and growth factors in the wound environment^2^. Besides these factors, acute infection, poor perfusion and nutrition and repetitive pressure also act as barriers to wound healing process^3^. Further, microbial infection on both acute and chronic wounds would result in the delay of healing process and impart severe infliction and associated secondary health problems^4^.

In order to eradicate the bacterial infection over the wound, a large variety of antibiotics and biocides are used. Even though planktonic bacteria can be effectively treated with antibiotics, the prolonged use of antibiotics substantially reduces the effectiveness of treatment, wherein the pathogens develop resistivity by forming a biofilm. In biofilm formation, in order to protect themselves, planktonic bacteria adhere strongly to the wound site and slowly develop into microcolonies through interaction with neighbouring microcolonies that emerge into a mature biofilm covered with extracellular polymeric substance (EPS)^5^ A matured biofilm protects bacteria from antibiotics by developing the tolerance to antimicrobials, environmental stress, inhibitors and chemical attack^6^. Hence, the bacterial biofilms on wound bed impairs the healing of both acute and chronic wounds.

Though a variety of strategies like mechanical debridement, application of extensive topical antimicrobials, wound cleansing, systemic application of antibiotics and antiseptics are usually employed to eliminate the impact of biofilm on the wound healing process, they are ineffective and have limitations^7^. For example, in case of frequent wound debridement, patients feel discomfort because of the pain and delayed healing due to the continuous disturbances in the wound bed^8^. This necessitates the need for an advanced strategy to treat bacterial biofilm-based infections. Recently, several antibiofilm agents such as lactoferrin, xylitol, gallium, dispersin B, acetylsalicylic acid, and numerous gel-based materials and silver alginate wound dressings have been developed to eliminate biofilms^9–13^. However, none of the above strategies proved to be efficient in suppressing or eliminating the biofilms, when used alone. Hence the development of multifunctional materials displaying effective wound closure and antibiofilm properties are required. In particular, biofilm formation is generally observed in diabetes patients undergoing wound closure treatment^14,15^ because of delayed improper hemostasis, extended inflammation, poor angiogenesis, fibroblasts, keratinocytes impaired migration and proliferation, faint vascularization, cellular infiltration, difficulty in re-epithelialization and connective tissue remodelling^14–17^. For diabetic wound closure, several clinical strategies such as topical application of growth factors, cellular therapies and biomaterial based scaffolds were used to regenerate skin and muscle^18–21^, which has several limitations such as uncontrolled release, short half-life, diffusion into other parts from the targeted site, high costs, and multiple dosages leading to undesirable side effects^22,23^. Therefore, a probable solution for the management of diabetic wound is to develop a multifunctional wound dressing material displaying anesthetic, antibiofilm and wound closure properties.

In the last few decades several commercial polymer-based gels such as Gelrin C, Mebiol Gel, Hystem hydrogel and Biogelx products, to name a few, have been developed for biomedical applications^24–26^. Various cross linking and conductive hydrogels for effective wound healing along with antibacterial activity have also been reported^27–31^. Researchers have been actively involved in the development of self-assembled low molecular weight gels for the effective tissue regeneration and wound closure applications, which could overcome the existing limitations associated with the polymer gels such as thermo-reversibility, processability, bio-compatibility, critical gelation concentration, sol–gel transition temperature (Tgel) and high molecular weight^32–36^. In this line, there is a great demand in the construction of multifunctional self-assembled gel-based materials for biomedical application, especially diabetic wound closure and infection management. In our previous report, we have developed an oleogel and composite gel from α-chloralose, a carbohydrate based molecule displaying long lasting anesthetic property^37^, which displayed good viscoelastic properties by molecular self-assembly of glycolipidand excellent wound healing activity in normal Wistar rat models^38^. In continuation of our previous work with promising results, we were curious to examine the wound closure behaviour of our self-assembled low molecular weight gels towards diabetic wounds and explore the antibiofilm property of sugar derived self-assembled gels^39–41^. In this regards, we report a multifunctional self-assembled glycolipid-based oleogel from FDA approved molecules, α-chloralose fatty acid and paraffin oil. The multifunctional anesthetic gel display potential diabetic wound closure and antibiofilm characteristics.

## Experimental section

### Materials and general methods

All essential chemicals, reagents and solvents used for the synthesis of glycolipids were purchased from Sigma Aldrich, Merck, TCI chemicals, Alfa aesar, SRL, and Avra chemicals. LR grade solvents were used for the compounds purification and AR grade solvents were used for synthesis and gelation studies. Distilled solvents were used, when necessary. Novozyme 435 was obtained from NOVOZYMES A/S, Denmark as a gift sample for our research purpose. The progress of the reactions was monitored by thin-layer chromatography (TLC) on pre-coated silica gel plates purchased from Merck and visualized by UV detection or using sulfuric acid spray or molecular iodine. Column chromatography was performed on Silica Gel (100–200 mesh) purchased from Avra chemicals, India^38^.

### Bacterial strains and culture conditions

Strains used in this work are Methicillin-resistant *Staphylococcus aureus* ATCC 43866, Methicillin-susceptible *Staphylococcus aureus* ATCC 25923, and *Pseudomonas aeruginosa* ATCC 27853. Lysogeny broth (LB) was used to maintain and grow the bacteria for most of the bacteriological experiments. However, biofilm experiments were performed with Tryptic Soy broth (TSB) for *S. aureus* and LB medium for *P. aeruginosa*. The absorbance for measuring the planktonic and biofilm growth was monitored in Tecan Sunrise™ Microplate Reader^38,40^.

### Planktonic and biofilm assay

Quantitative planktonic growth and biofilm development were measured by the method described by O’Toole and Kolter^42^. Briefly, an overnight grown culture was diluted to 1:100 and inoculated in 200 μL of the medium containing different concentrations of the glycolipids in the microtiter well and was incubated for 24 h at 37 °C^40^. The absorbance of each well was measured at 595 nm using the microplate reader for the planktonic growth. Then the grown culture was aspirated, and the wells were washed three times with 200 μL of sterile phosphate-buffered saline (PBS) to remove the non-adherent cells. Wells were then dried, and the adherent biofilm was stained with 200 μL of 0.1% crystal violet (CV) for 15 min. The unbound CV was removed by rinsing thrice with 200 μL of PBS. Further, 70% ethanol was added and incubated for 15 min to de-stain the CV and the absorbance of each well was measured at 595 nm using the microplate reader as a proxy for the biomass^38^.

For fluorescent microscopic analysis of the biofilms, cells were grown on glass slides in a petri plate containing broth (LB/TSB) with DMSO/Compounds of concentration 400 μg mL^−1^ at 37 °C for 24 h. Then the slides were washed thrice with PBS and biofilm was stained with SYTO9 in dark condition. Slides were then observed under the fluorescence microscope (Nikon Eclipse Ni–U). Multiple images (minimum 20 images) were taken and processed with auto-thresholding technique and the intensity was measured using the ImageJ software.

### In vivo* wound closure studies and ethical issues*

Healthy Wistar strain female rats (200–230 g) of 8–9 weeks old were procured from Central Animal Facility, SASTRA Deemed University, Thanjavur, Tamil Nadu, India. The animals were housed individually in the standard laboratory environment for 7 days. During the experiment, the rats were fed with standard pellet diet and water ad libitum. The experimental protocols used in this study were approved by the Institutional Animal Ethics Committee (IAEC), SASTRA Deemed University and experiments were carried out as per the guidelines of Committee for Control and Supervision of Experimental Animal (CPCSEA), New Delhi (Reg. No. 817/04/ac/CPCSEA), SASTRA Deemed University, Thanjavur, India and its approval number is 442/SASTRA/IAEC/RPP^38^.

### Diabetic induction

Rats were fasted overnight and then induced by intraperitoneal injections of streptozotocin (STZ) dissolved in sodium citrate buffer (0.1 M, pH 4.5) at a dosage of 65 mg/kg body weight. Blood glucose levels were monitored by a glucose meter (On-call plus, USA) one week after induction of diabetes. Blood was drawn from the tail vein and glucose levels were determined. Rats with blood glucose levels > 250 mg/dL were considered as diabetic rats^43^.

### Experimentally inflicted wounds

Diabetes-induced Wistar strain female rats were randomly segregated into five groups comprising six rats each and caged individually. The animals were anesthetized by thiopentone sodium (60 mg/Kg, i.p) before the wound creation. The rats were inflicted with excision wounds. The dorsal fur of the animals was shaved, and the anticipated area of the wound created was marked. A full-thickness excision wound was created by existing 200 mm^2^ areas of skin in length and 0.2 cm depth from the dorsal region using a sharp surgical blade and pointed scissors^38^.

### Topical application of gels

The wound of Group 1 rats was kept as such without any treatment, and this group served as the control, Group 2 rats were treated with 0.1 mL of paraffin oil and this is served as vehicle control. The wound of Group 3 and Group 4 was treated with 0.1 mL of oleogel **1** (2% w/v) and oleogel **2** (4% w/v) respectively. Group 5 was treated with Composite gel (2% w/v). For the studies, 0.1 mL of the gel was applied on the wound surface once a day and monitored the wound closure every day^38^.

### Estimation of the rate of wound contraction

Progress of wound closure was monitored by tracking the wound area on a transparent graph sheet on alternate days up to 21 days for all six animals in each group. The transparent graph sheet was laid over the wound and traced it using a permanent marker. The area of wound size was studied by using ImageJ software. The diameter was recorded and the percentage of closure of the wound was calculated until the day of complete epithelialization. The standard and mean deviations were given in sq.mm. Percentage of wound contraction was calculated using the initial area and the area under investigation:

$$\% \,{\text{of wound contraction}} = \left[ {\left( {W_{1} - W_{0} } \right)/W_{1} } \right] \times 100$$

where *W*_1_ and *W*_0_ represent the initial wound size and wound size of the specific day respectively^38^.

On the 21st post wounding day, the animals were sacrificed, blood samples and granulation tissue samples were collected for the estimation of free radicals^44^, ascorbic acids^45^, hexosamine^46^, and collagen tissue parameters^47^.

### Statistical analysis

All values were reported as the mean ± error bars that either indicate SEM or SD or CI at 95%, which is mentioned in the legends accordingly Significance was measured by either *t* tests for comparison of two samples and one-way ANOVA with appropriate post hoc tests for multiple samples, which is also mentioned in the legends. A value of *P* < 0.05 was considered significant. Statistical analysis was performed using GraphPad Prism for Windows software^38^.

### Histopathological examination

Wound tissue specimens from control and test groups were taken after complete healing of excision wound and after usual processing skin samples were cut a thickness of 6 µm and stained with hematoxylin and eosin (H&E). Sections were qualitatively assessed under the light microscope and the results were recorded^38^.

## Results and discussion

### Synthesis, gelation, morphological and rheological studies

**Scheme 1:**
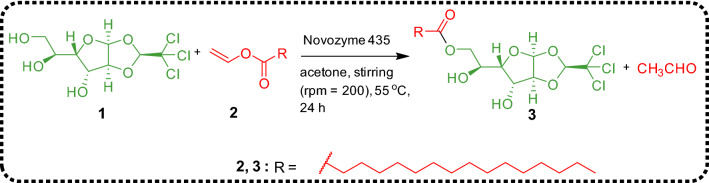
Synthesis of anesthetic glycolipid **3**.

Glycolipids are a type of biological lipids found widely in plant and animal systems, performing various brilliant tasks. Generally, glycolipids consist of hydrophilic carbohydrate moiety covalently attached with hydrophobic fatty acids or any other source via glycosidic bond. In the present studies, glycolipid **3** was synthesized by the enzymatic transesterification of α-chloralose with vinyl palmitate using Novozyme 435 by following the procedure described in our previous report (Scheme 1)^38^. The synthesized glycolipid was characterized by NMR and mass spectral techniques^38^. The glycolipid **3** displayed gelation in eucalyptus oil (1% w/v), 1: 4 DMSO + Water (2.5% w/v) and paraffin oil (1% w/v). In the present investigation, we have chosen an oleogel formed by **3** in paraffin oil via molecular self-assembly guided by intermolecular non-covalent interactions because of broad range of applications displayed by paraffin oil in cosmetics, food and pharmaceutical industries^38^. In addition to the oleogel, we have prepared composite gel by encapsulating the natural drug, curcumin within the oleogel matrix.

**Figure 1:**
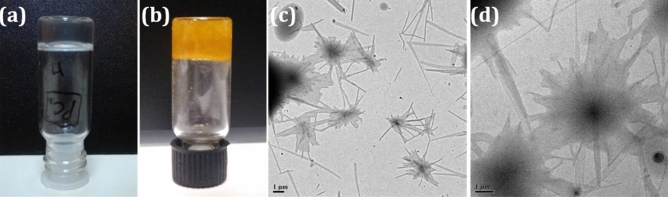
(**a**,**b**) Images of oleogel and composite gel in paraffin oil; (**c**,**d**) HRTEM images of oleogel and composite gel.

Morphological analysis of both oleogel and composite gel was investigated by HRTEM analysis. In both the cases the formation of entangled fibrillar network with 200–500 nm thickness is observed (Fig. 1), which implies that after incorporation of curcumin, there is no drastic change in the morphology of gel. However, in composite gel, the influence of curcumin in the gel network could be identified by rheological measurements. Rheological studies of the oleogel and the composite gel confirmed their thermoreversible, thermal stability and thixotropic nature (Figs. S1–S3)^38^. Both oleogel and composite gel were stable under physiological conditions.

Rheological studies clearly establish the injectable nature of both oleogel and composite gel derived from glycolipid **3**, which would help in wound closure by regulating the overlapping phases. However, in both traumatic or surgical injury, the healing of wound is delayed due to the microbial contamination, particularly in diabetics. This phenomenon motivated us to search for new materials with the antimicrobial, antibiofilm and tissue regenerating properties.

### Antibacterial studies

**Figure 2:**
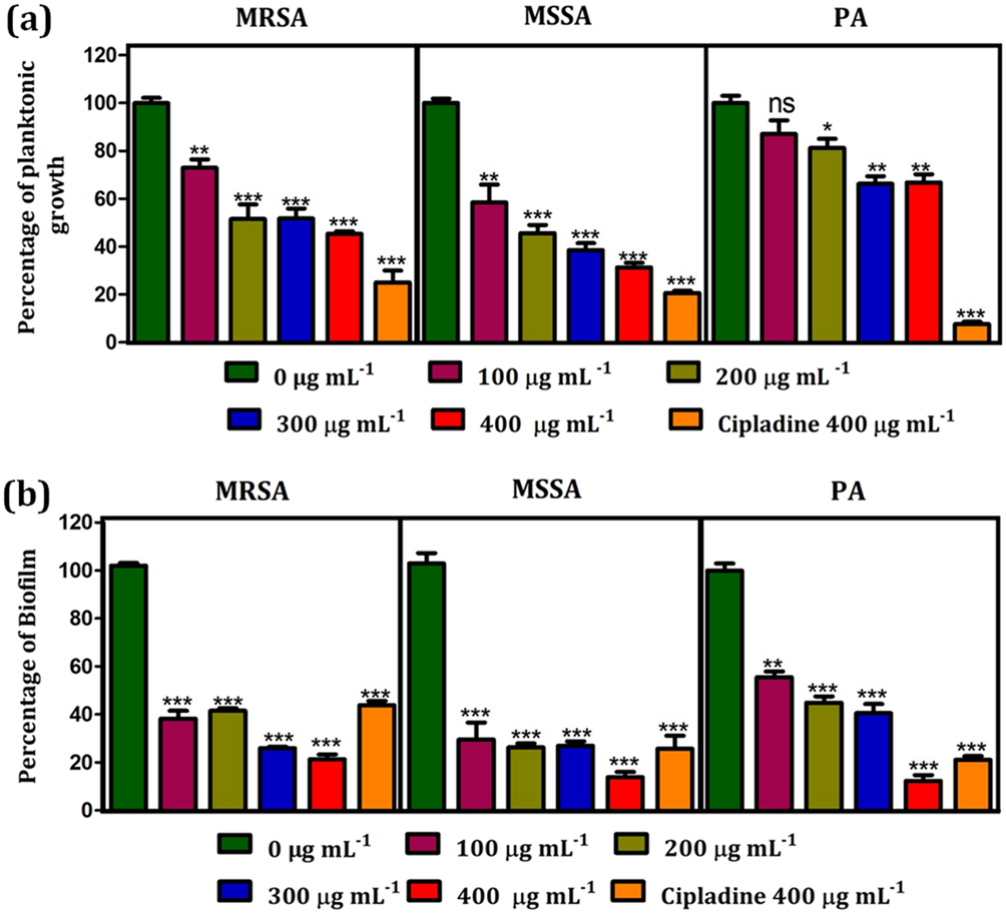
Influence of the different concentrations of glycolipid **3** on (**a**) bacterial cell viability and (**b**) biofilm formation. The bars represent the bacterial growth and biofilm biomass at an absorbance of 595 nm. Error bars represent 95% CI, *n* = 4. One-way ANOVA was performed to analyze the data. Significance between 0 μg mL^−1^ with other concentrations is depicted (*P < 0.05, **P < 0.01, ***P < 0.001).

In vitro antibacterial properties of glycolipid **3** were investigated against various wound infection-causing pathogenic bacteria namely Methicillin-resistant *Staphylococcus aureus* (MRSA), Methicillin-susceptible *Staphylococcus aureus* (MSSA) and *Pseudomonas aeruginosa* (PA). The influence of various concentrations of the glycolipid **3** on bacterial growth were studied. It is observed that the glycolipid **3** inhibited the growth of MRSA and MSSA at concentrations 200 μg mL^−1^, 300 μg mL^−1^ and 400 μg mL^−1^ (*P* < 0.001). In the case of PA, growth was not reduced, however, a mild inhibition of 20 and 25% was observed at 300 μg mL^−1^ and 400 μg mL^−1^ (*P* < 0.01) respectively, while the standard control drug, cipladine inhibited more than 75% growth of all organisms at 400 μg mL^−1^ (*P* < 0.001). Among these three pathogens tested, the glycolipid **3** displayed a substantial antibacterial effect on both MRSA and MSSA (Fig. 2a). The MIC of the glycolipid **3** on planktonic growth is shown in Table 1.

**Table 1:** The minimum inhibitory concentration of glycolipid **3** on planktonic growth and biofilm formation.

Bacterial species	MIC^a^ (µg mL^−1^)	MBIC^b^ (µg mL^−1^)
Methicillin-resistant *S. aureus*	400	300
Methicillin-susceptible *S. aureus*	300	200
*Pseudomonas aeruginosa*	ND	300

*ND* not determined.

^a^Minimum inhibitory concentration.

^b^Minimum biofilm inhibitory concentration.

### Anti-biofilm studies

After exploring the antibacterial activity of the glycolipid **3** against various wound infection-causing pathogenic bacteria, the anti-biofilm activity of **3** towards different pathogenic bacteria was investigated by varying the concentrations (Fig. 2b). The glycolipid **3** inhibited MRSA, MSSA, and PA at all concentrations (*P* < 0.001). Surprisingly, at 400 µg mL^−1^, glycolipid **3** displayed more antibiofilm activity than the standard drug cipladine. These results clearly showed that the mode of action of the drugs was different in planktonic and biofilm form of microbes. The minimum inhibitory concentration required to inhibit the biofilm formation by glycolipid **3** is 200 µg mL^−1^ for MSSA and 300 µg mL^−1^ for both MRSA and PA (Table 1).

**Figure 3:**
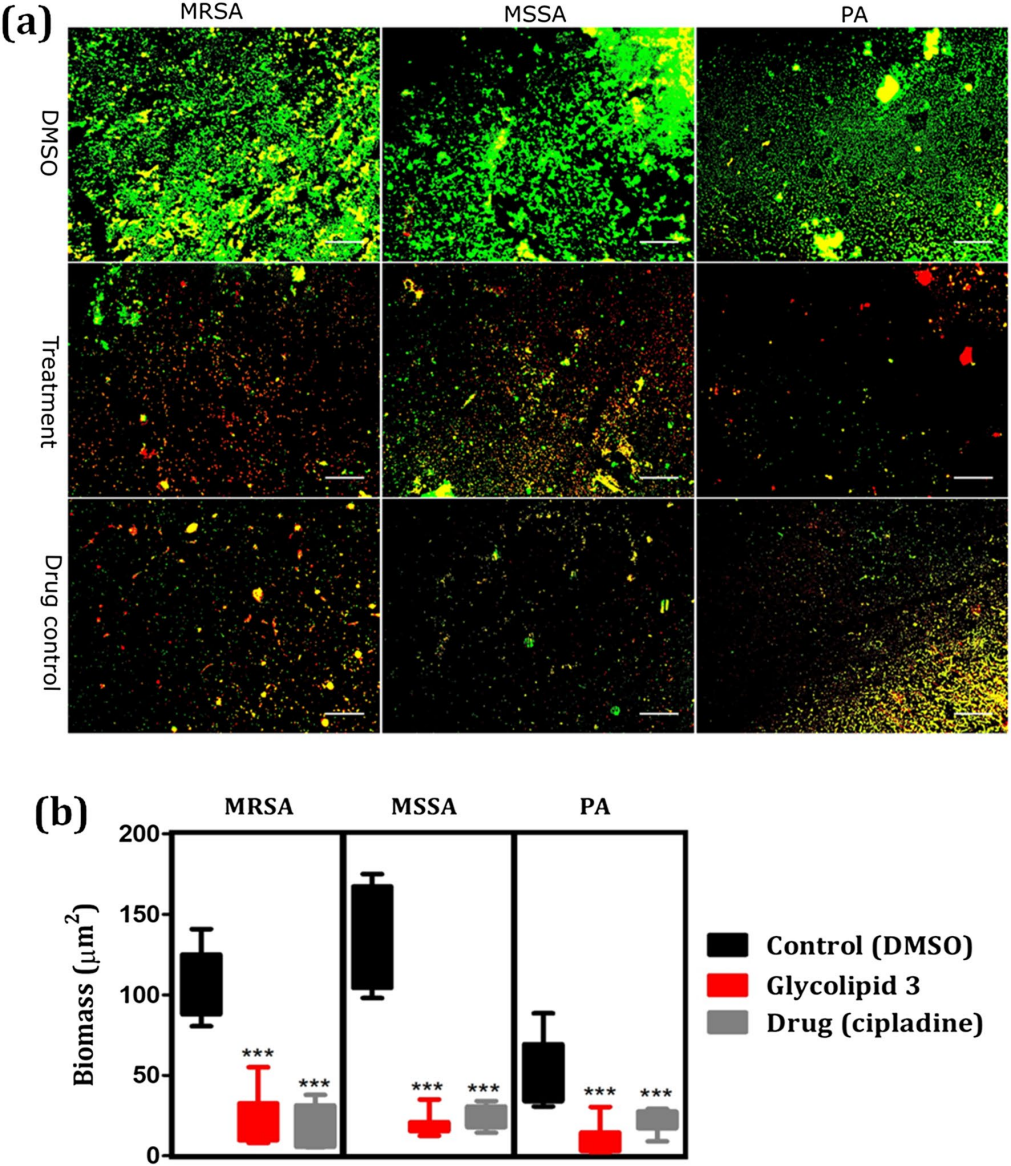
(**a**) Representative fluorescence images of the biofilms treated with DMSO (control), glycolipid **3** (treatment) and drug control (cipladine). Biofilm formed by MRSA, MSSA, and PA was stained with SYTO9 and PI. Green colour represents live cells and red represents the dead cells. Scale bar is 250 µm; (**b**) Quantification data obtained from fluorescent microscopic images of biofilms formed from MRSA, MSSA and PA. *N* =  > 20. Mann–Whitney *U* test was performed to analyze the data (***P < 0.001).

Further, the influence of glycolipid **3** on biofilm formation was confirmed by using fluorescence microscopy. Biofilm formation was monitored on glass slides that were grown on broth (LB or TSB) containing DMSO and 400 μg mL^−1^ of glycolipid **3** or standard drug, cipladine as a drug control. The dense matured biofilm formation with several microcolonies fused with voids was observed in control slides for all three pathogens (Fig. 3a). Compared to DMSO and drug control, glycolipid **3** displayed the inhibition of the formation of MRSA, MSSA, and PA biofilms to a greater extent (Fig. 3a). The quantification of the images also revealed the significant reduction of the biofilm biomass by the glycolipid **3** (Fig. 3b).

**Figure 4:**
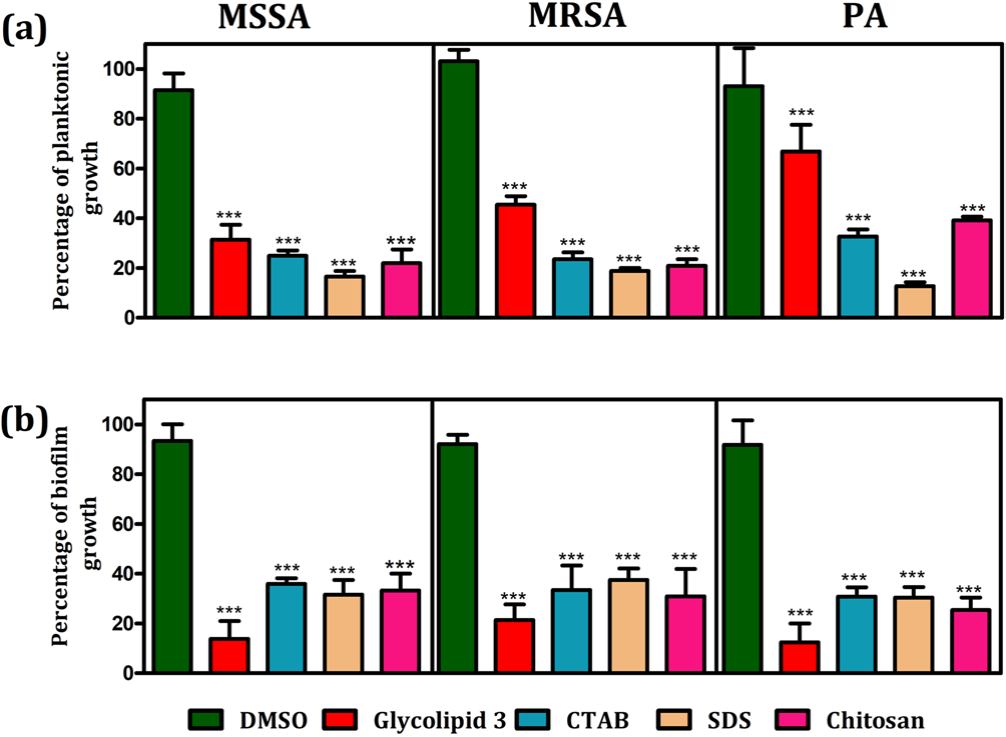
Influence of glycolipid **3** on (**a**) planktonic growth and (**b**) biofilm formation in comparison with CTAB (a cationic surfactant), SDS (an anionic surfactant), chitosan, and DMSO (vehicle control) on MSSA, MRSA, and PA. Error bar indicates 95% CI, *n* = 4, one-way ANOVA with Tukey’s test was used to determine significance (***P < 0.001).

The chronic wound infections are usually caused by bacteria in its biofilm lifestyle^48^. The microbial clusters enmeshed in matrix components are defined as biofilms. The cells lose the typical biofilm characteristics devoid of matrix. Therefore, we hypothesized that the glycolipid **3** might repress the matrix production in the cultures thereby inhibiting its biofilm. As higher levels of intracellular c-di-GMP determines matrix production, we used the fluorescent biomarker of *P.aeruginosa* to identify the effect of glycolipid **3** on the levels of c-di-GMP^49^ Sodium nitroprusside that produces nitric oxide, which acts as a signalling molecule to reduce c-di-GMP levels was used as a positive control^50^. The glycolipid **3** did not influence the intracellular c-di-GMP levels in *P. aeruginosa* indicating that the matrix production may possibly not be intervened by it through this signalling pathway (Fig. S4). We tested the effect of glycolipid **3** on matrix production by Congo red (CR) plate assay, where the matrix components bind to CR and turns the Staphylococcus colony colour into black^51^. The MSSA and the MRSA colonies did not show any change in the colony colour or the morphology indicating that the glycolipid **3** did not impair the matrix production to inhibit biofilm formation (Fig. S5). We further hypothesised that the glycolipid **3** possibly inhibited biofilm by altering the surface similar to other surfactants. Biosurfactant production by the bacteria is a strategy to disperse from the biofilm cells and it aids in the emergence of typical biofilm structure^52^. Surfactants are also well known to display antibiofilm activity. We determined the equivalent concentration of cationic surfactant, CTAB, an anionic surfactant, the SDS, and chitosan (a known cell permeabilizing agent) to the permeablizing activity of glycolipid **3** (Fig. S6). We then used these compounds in appropriate concentrations with more or less similar permeabilization index (Fig. S6) to test the biofilm inhibitiory activity of the organisms. We observed that the planktonic growth as well as the biofilm formation was significantly inhibited by the compounds used (Fig. 4). This indicates that cell permeabilization is the possible mechanism for inhibiting planktonic growth, and the alteration of the cell surface or biofilm matrix could be the mechanism for biofilm inhibition. However, the glycolipid **3** was more effective in biofilm inhibition in all the three organisms than the planktonic growth inhibition (Fig. 4). Surfactant-based polymer dressings have also been reported to disrupt biofilm matrix in *P. aeruginosa* and *S. aureus*^53^.

### In vivo* wound closure studies*

**Figure 5:**
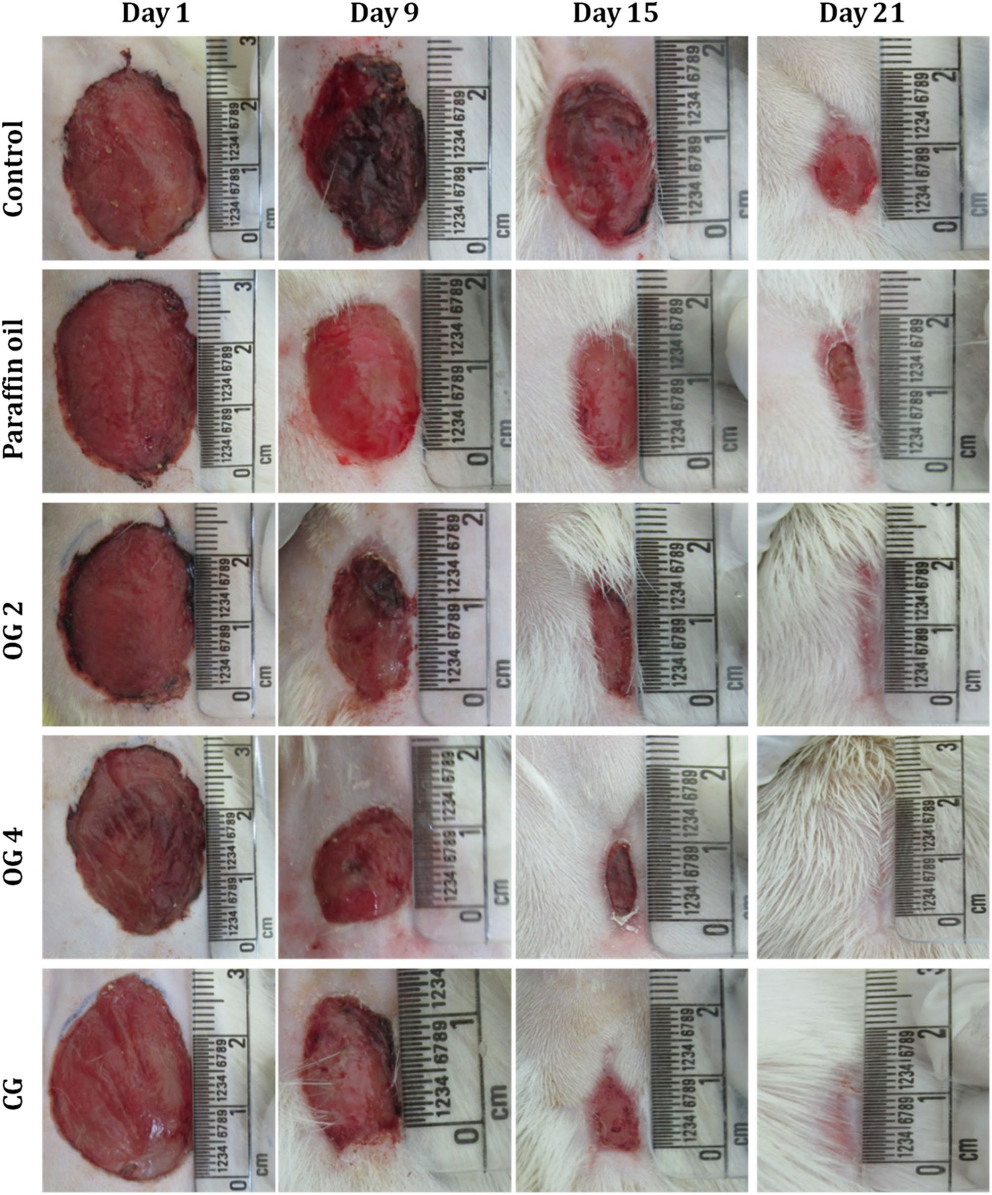
The comparative assessment of the gross appearance of wound healing on different treated groups for different experimental days 1, 9, 15 and 21 in diabetes induced Wistar rats. OG 2 (oleogel 1) and OG 4 (oleogel 2) represents oleogel of 2% w/v and 4% w/v respectively. CG denotes composite gel of 2% w/v.

In our early work, we have demonstrated the wound closure ability of oleogel and composite gel in normal Wistar rat models. Since antibacterial and antibiofilm results were highly promising, we were curious to investigate the wound closure behavior of oleogel and composite gel in diabetic rats. In this investigation, we have used Oleogels and composite gel derived from glycolipid **3** and paraffin oil as vehicle control, and one group is left without any treatment, which is considered as a control group. Figure 5 clearly reveals that on the 21st day, 98% and 97% of wound healing were observed in diabetic rats treated with oleogels and composite gel respectively. Oleogel treated animals displayed dose-dependent wound closure from day 3 onwards indicating progressive wound healing. On the 15th day, more than 85% of the wound contraction was seen in oleogel and composite gel treated rats (Fig. S7, Table 2).

**Table 2:** Effect of oleogel and composite gel on the percentage (%) of wound healing in experimental rats.

Group	Percentage of wound healing (mean + SEM)			
	Day 3	Day 9	Day 15	Day 21
Control	5.62 ± 1.27	39.402 ± 0.772	68.208 ± 0.544	85.97 ± 0.46
Paraffin oil	6.34 ± 1.55	54.56 ± 0.62**	76.89 ± 0.625*	90.91 ± 0.135*
Oleogel **1**	10.04 ± 1.30	64.26 ± 0.62***	86.60 ± 0.44**	96.05 ± 0.19**
Oleogel **2**	12.94 ± 0.88*	72.91 ± 0.33***	90.60 ± 0.32***	98.92 ± 0.14**
Composite gel	12.83 ± 0.50*	68.21 ± 1.07***	87.39 ± 0.35**	97.06 ± 0.069**

^a^Percentage value of mean ± SEM of each group. Significance was *P* < 0.05 (*), *P* < 0.01 (**), *P* < 0.001 (***). Comparison of treated groups with the control group. The results were analyzed statistically using one-way ANOVA followed by Dunnett’s post-hoc test for multiple comparisons.

Generally, wound healing is a delayed process in diabetic patients due to poor angiogenesis, lack of oxygen and nutrient supply^32^. In this context, improving revascularization in the wound area is essential to improve the wound closure. Currently, various scaffolds combined with growth factors are used to promote angiogenesis in wound healing and tissue engineering applications^34,54,55^. In this study, oleogel and composite gel displaying 3-dimensional fibrous network formed via various intermolecular interactions could have served as a scaffold for wounds, which in turn possibly enhanced cell–cell communication, increased collagen synthesis, cell migration and promoted angiogenesis.

Further, the wound healing property of the gels was investigated in the aspect of expressing the biochemical profiles like collagen expression and stabilization, the proliferation of fibroblasts and lipid peroxidation. Generally, wound closure involves the migration and proliferation of endothelial cells^44,56^. We have discussed the importance of collagen in hemostasis and epithelization at the later phase of the wound healing in our earlier work^38^. Compared to control group (10.20 ± 1.03 mg/100 mg), the oleogel **1**, **2** and composite gel treated groups displayed an increased amount in hydroxyproline of 20.59 ± 1.56, 24.95 ± 1.97 and 21.88 ± 1.50 mg/100 mg respectively. Rapid formation of granulation tissues and increased synthesis of collagen around the wound area can be attributed to the enhancement of the wound healing rate with oleogel **2** and composite gel treated group (Fig. S8, Table 3). Vehicle group also showed increased amount of hydroxyproline compared to control group this is due to the medicinal properties of paraffin oil. From long time people has been using paraffin oil for various health issues^57–59^.

**Table 3:** Biochemical profile of granulation tissue obtained from the skin-excised wound of different experimental groups.

Group	Hydroxyproline (mg/100 mg)	Hexosamine (mg/100 mg)	Vitamin C (mg/100 mL)	Lipid peroxide (mg/100 mg)
Control	10.20 ± 1.03	0.55 ± 0.03	2.44 ± 0.07	4.86 ± 0.42
Paraffin oil	14.23 ± 1.03	0.72 ± 0.04*	2.53 ± 0.05	4.12 ± 0.20
Oleogel **1**	20.59 ± 1.56***	1.27 ± 0.05***	3.28 ± 0.09***	2.16 ± 0.08***
Oleogel **2**	24.95 ± 1.97***	1.41 ± 0.04***	3.93 ± 0.12***	1.26 ± 0.05 ***
Composite gel	21.88 ± 1.50***	1.38 ± 0.04***	3.98 ± 0.14***	1.29 ± 0 04***

Values of mean ± SEM of each group. *P* < 0.05 (*), *P* < 0.01 (**), *P* < 0.001 (***). Comparison of treated groups with a control group. The results were analyzed statistically using one-way ANOVA followed by Dunnett’s post-hoc test for multiple comparisons.

Glycosaminoglycans are one of the major components of the extracellular matrix of the skin, which exhibits viscoelastic and hygroscopic properties that are responsible for normal dermal tissue function. Hexosamine is a component of glycosaminoglycans, , which strengthens the collagen fibers by molecular interactions that manifests in its assembly. In oleogel **2** (1.41 ± 0.04) and composite gel (1.38 ± 0.04), treated groups display higher level of hexosamine, which further stabilized collagen via assembly process and facilitated the production of new extracellular matrix (Fig. S8, Table 3). Ascorbic acid commonly known as Vitamin C plays a major role in all phases of wound healing, it is an essential constituent that displayed antioxidant property, facilitate the blood vessel repair and activation of dermal fibroblasts in the tissue regeneration process. In the present study, increased levels of ascorbic acid have been observed in the groups treated with the oleogel **2** and composite gel (3.93 ± 0.12 and 3.98 ± 0.14 respectively) compared to the other groups (Fig. S8, Table 3). Increased levels of ascorbic acid in this groups are evidence of effective wound healing. Hydroxyl radical produced in the wound area stimulates the lipid peroxidation that affects the healing process by causing damage to proteins, lipids, DNA, cellular membranes, and fibroblast metabolism^60^. However, the topical application of oleogel **2** and composite gel treated groups decreased the levels of lipid peroxide 1.26 ± 0.05 and 1.29 ± 0.04 respectively (Fig. S8, Table 3). Lipid peroxidation result revealed the formation of collagen fibrils and the activation of various enzymes that prevents cell damage. Inhibition of lipid peroxidation levels also induced the Vascular Endothelial Growth Factor (VEGF) expressions^61^ favoring the wound contraction in diabetic wounds.

**Figure 6:**
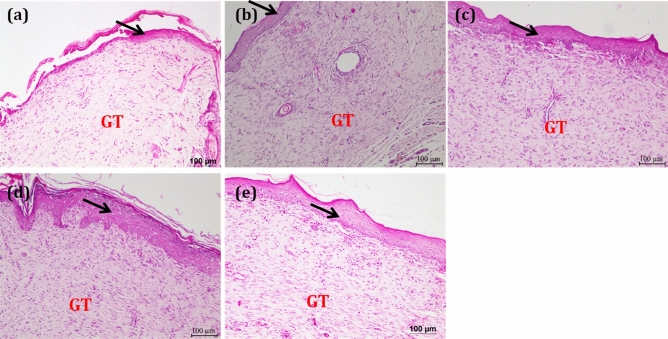
Haematoxylin and Eosin (H&E) stained sections of healed skin of experimental groups (**a**) Control group shows moderate epithelialization with early wound contraction phase, (**b**) vehicle control paraffin oil-treated skin section shows moderate epithelialization with mid wound contraction phase, (**c**) oleogel **1-** treated group showing granulation tissue with moderate epithelialization with late fibroblast phase, (**d**) oleogel **2—**treated group showing granulation tissue with complete epithelialization, (**e**) composite gel displaying complete epithelialization at 10X magnifications. Arrow—Epidermis; GT—Granulation tissue.

Further, histopathological studies have been performed to investigate the wound healing rates of various treated groups. The hematoxylin and eosin-stained skin sections of various treatment groups displayed different wound healing rates (Fig. 6). Control groups shows moderate epithelialization with early and mid wound contraction phase respectively, where as in oleogel and composite gel treated groups shows complete epithelialization with late wound contraction phase. Thus histological data also reveals the high degree of wound healing in oleogel and composite gel treated groups compare to control groups The topical application of oleogel **2** and composite gel displayed effective tissue regeneration when compared to other groups without any edema and congestion.

## Conclusions

We have synthesized anesthetic glycolipid from FDA approved starting materials, α-chloralose and palmitic acid using Novozyme 435 as a biocatalyst. Supramolecular self-assembly of glycolipid in paraffin oil using various intermolecular interactions furnished oleogel, which form 3-dimensional fibrous architecture identified using HRTEM analysis. In medicine, paraffin oil is commonly used as an additive in pediatric laxative, cosmetic products, and to treat arthritis, fibromyalgia and dry skin, hence, we have explored the application of oleogel and composite gel prepared by encapsulating curcumin into the gel matrix in skin wound closure. In this report, we present a multifunctional gel material displaying anesthetic, antibacterial, antibiofilm and skin wound closure for traumatic and surgical injury. The antibiofilm property of anesthetic glycolipid was investigated over common dermal infection-causing bacteria, Methicillin-resistant *Staphylococcus aureus*, Methicillin-susceptible *Staphylococcus aureus*, and *Pseudomonas aeruginosa* and found to be better than standard drug cipladene at 400 μg mL^−1^. Both oleogel and composite gel displayed enhanced skin wound closure in diabetic Wistar rats. The reported multifunctional gel-based material will surely open up a new avenue in biomedical field, especially for diabetic patients suffering from traumatic and surgical injury.

### Supporting information

Supplemenary methods, rheology, wound healing studies, biochemical profile, C-di-GMP study, CRA assay, and cell permeability studies.

## Supplementary information

Supplementary file1

## Abbreviations

ECM: Extracellular matrix
EPS: Extracellular polymeric substance
FDA: US food and drug administration
TLC: Thin-layer chromatography
LB: Lysogeny broth
TSB: Tryptic soy broth
PBS: Phosphate-buffered saline
MRSA: Methicillin-resistant *Staphylococcus aureus*
MSSA: Methicillin-susceptible *Staphylococcus aureus*
PA: *Pseudomonas aeruginosa*
MIC: Minimum inhibitory concentration
MBIC: Minimum biofilm inhibitory concentration
VEGF: Vascular endothelial growth factor
