# Supplementary material for: An injectable self-healing anesthetic glycolipid-based oleogel with antibiofilm and diabetic wound skin repair properties
Source: Sci Rep. 2020 Oct 22;10:18017. doi: 10.1038/s41598-020-73708-7 (PMC7582191; doi:10.1038/s41598-020-73708-7)
Supplement: Supplementary file 1 — Supplementary file1 [file 41598_2020_73708_MOESM1_ESM.docx]

**An Injectable Self-Healing Anesthetic Glycolipid-Based Oleogel with Antibiofilm and Diabetic Wound Skin Repair Properties**

Yadavali Siva Prasad,^a^ Sandeep Miryala,^b^ Krishnamoorthy Lalitha,^a^ Balasubramani Saritha,^a^ C. Uma Maheswari,^a^ Vellaisamy Sridharan,^c^ C. S. Srinandan*^b^ and Subbiah Nagarajan*^a,d^

^a^ Organic Synthesis Group, Department of Chemistry, School of Chemical and Biotechnology, SASTRA Deemed University, Thanjavur – 613401, Tamil Nadu, India.

^b^ Biofilm Biology Lab, Centre for Research in Infectious Diseases, School of Chemical and Biotechnology, SASTRA University, Thanjavur-613401, Tamil Nadu, India.

^c^ Department of Chemistry and Chemical Sciences, Central University of Jammu, Rahya-Suchani (Bagla), District-Samba, Jammu-181143, Jammu and Kashmir, India

^d^ Department of Chemistry, National Institute of Technology Warangal, Warangal -506004, Telangana, India.

Corresponding authors:

Subbiah Nagarajan - Tel: +91-9940430715; E-mail: snagarajan@nitw.ac.in

C. S. Srinandan - Tel: +91 4362 304270; E-mail: srinandan@gmail.com

Table of contents

1. Supplementary methods 3
2. Figure S1 Strain amplitude and Angular frequency dependence of G′ and G″ of Injectable oleogel (OG) and composite gel (CG) 5
3. Figure S2 Temperature dependence of G′ and G″ of oleogel and composite gel 5
4. Figure S3 Thixotropy-loop test via continuous step-strain measurements of oleogel 5
5. Figure S4 Influence of glycolipid **3** on the levels of cyclic-di-GMP 6
6. Figure S5 Influence of glycolipid **3** on biofilm matrix production by Congo Red plate assay 6
7. Figure S6 Cell permeability profile by glycolipid **3** and other commonly known surfactants 7
8. Figure S7 Effect of oleogel and composite gel on wound healing in diabetic rats 8
9. Figure S8 Biochemical proﬁle of granulation tissue obtained from the skin-excised wound of diﬀerent experimental groups 9
10. Figure S9 ^1^H NMR spectrum of glycolipid **3** in CDCl_3_+DMSO-d_6_ 10
11. Figure S10 ^13^C NMR spectrum of glycolipid **3** in CDCl_3_+DMSO-d_6_ 11
12. Figure S11 ESI-MS spectra of glycolipid **3** in CDCl_3_+MeOH 12
13. References 12

**Supplementary Methods**

**Synthesis of oleogelator, 3**. To a stirred solution of α-chloralose (1 mmol) in acetone (5 mL), vinyl ester (3 mmol) and Novozyme 435 (100 mg) was added. The entire contents were kept in orbital shaker incubator at 55 ^o^C and stirred at 300 rpm. The progress of the reaction was monitored by using thin layer chromatography and after completion of the reaction, the entire mixture was cooled to room temperature and filtered to remove Novozyme 435. The residue in the Buchner funnel was washed well with acetone. The filtrate was concentrated by using a rotary evaporator at reduced pressure. Further the residue is diluted with DCM and washed well with water. The organic layer was extracted and dried over Na_2_SO_4,_ concentrated to obtain whitish crude solid. The crude residue was purified by silica gel column chromatography using hexane- ethyl acetate as eluent.^1^

***C-di-GMP reporter assay***

We used the *P. aeruginosa* strain and procedure according to Rybtke et al., to test the influence of glycolipid **3** on the intracellular levels of c-di-GMP.^2^ The *P. aeruginosa* strain was streaked on ABTG agar supplemented with gentamicin and inoculated into a test tube with ABTG + casA supplemented with gentamicin. The overnight grown culture was diluted to 0.03 at an OD of 600 nm into 100 ml ABTG + casA supplemented with gentamicin and grown for an additional 24 h. As controls we added sodium nitroprusside (SNP) that produces nitric oxide to a concentration of 250 µM, and DMSO was used as a vehicle control. Growth of the culture was analysed as biomass at OD 600 nm, and the green fluorescence was measured to ascertain the intracellular level of C-di-GMP. Fluorescence was measured as arbitrary fluorescence intensity units on a Hybrid Multi-Mode Reader (Synergy H1 BioTek plate reader) using a 490-nm narrow-band excitation filter, a 515-nm sharp-cut emission filter. Relative Fluorescent Units (RFU) were calculated as the ratio of arbitrary fluorescence intensity units to cell biomass as determined by the absorbance at 600 nm.

***Congo red agar assay***

The colony morphology of the Staphylococcal strains was tested by adding 40 µg mL^-1^ of Congo red dye and 20 µg mL^-1^ of Coomassie brilliant blue to tryptic soy agar. This assay was used for qualitatively determining the matrix production.^3,4^

***Measuring the permeability of the cells***

The permeability of the cells was analysed using propidium iodide (PI) fluorescent dye.^4,5^ The overnight grown culture was harvested at 10000 g for 10 min and washed thrice with sterile PBS. Supernatant was discarded and the pellet was resuspended with equal volume of PBS and 5 µL of PI was added in all controls and test samples. Here negative control was, the cells and 5 µL PI devoid of any surfactants. For test samples, appropriate concentration of the glycolipid **3**, SDS, CTAB, and chitosan were added along with PI and incubated for an hour. The fluorescence was measured using in the multimode reader using a 500-nm excitation filter, a 600-nm emission filter. Permeability index was calculated by normalizing the fluorescence value of the test samples to that of the negative control.


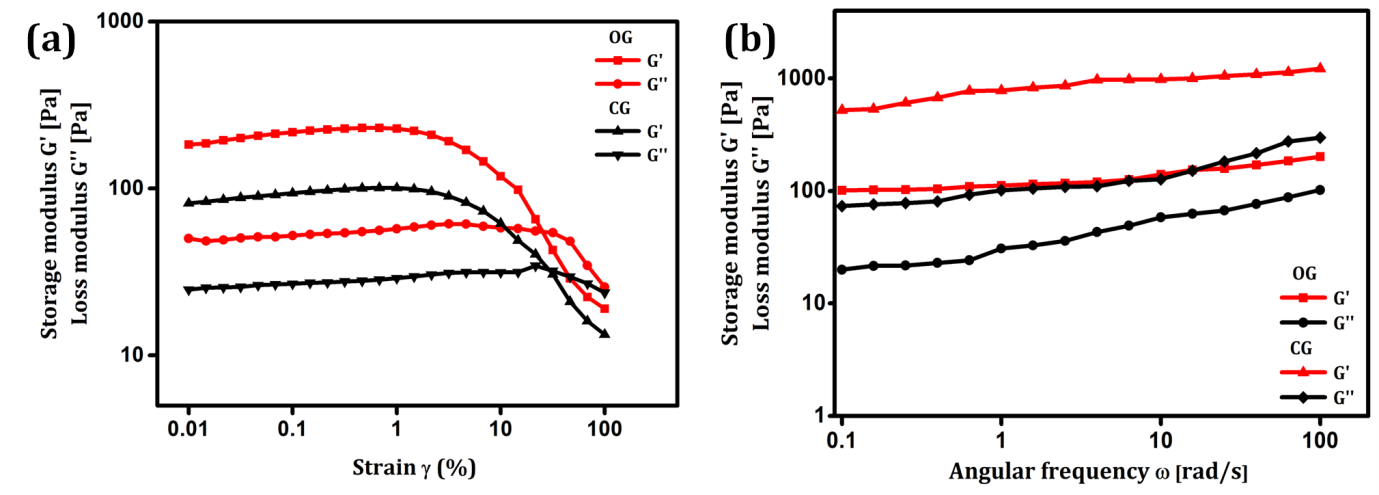


**Figure S1.** (a & b) Strain amplitude and Angular frequency dependence of G′ and G″ of Injectable oleogel and composite gel respectively.


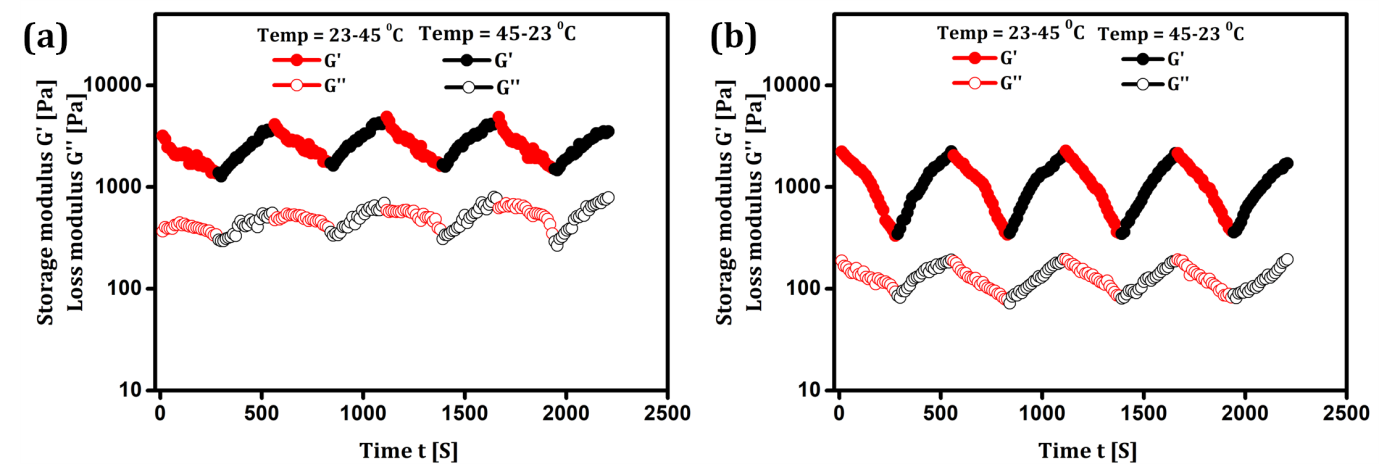


**Figure S2** (a & b) Temperature dependence of G′ and G″ of oleogel and composite gel respectively.

**
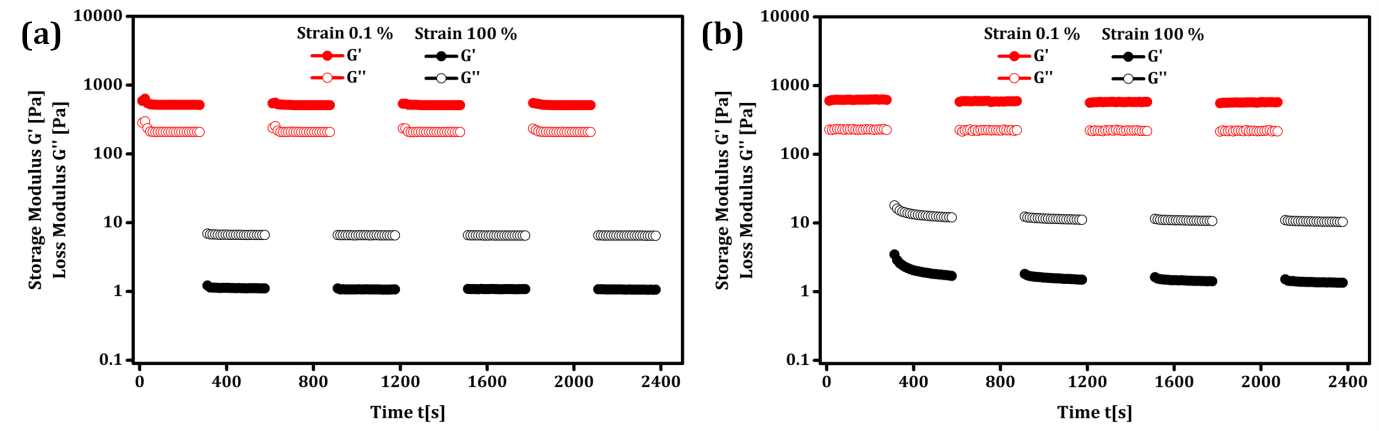
**

**Figure S3.** (a & b) Thixotropy-loop test via continuous step-strain measurements of oleogel and composite gel.

**Figure S4**. Influence of glycolipid **3** (Test) on the levels of cyclic-di-GMP. Sodium nitroprusside (SNP) is the positive control and DMSO is the vehicle control. Relative Fluorescence Units (RFU) represent the arbitrary fluorescence intensity units that is corrected
for cell density. *n* = 4.


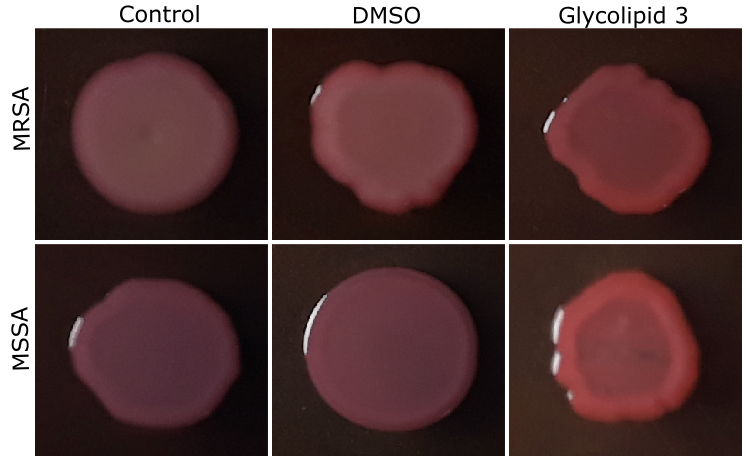


**Figure S5**. Influence of glycolipid **3** on biofilm matrix production in the Methicillin sensitive and resistant types of *Staphylococcus aureus* as visualized by its colony morphology on Congo red agar.


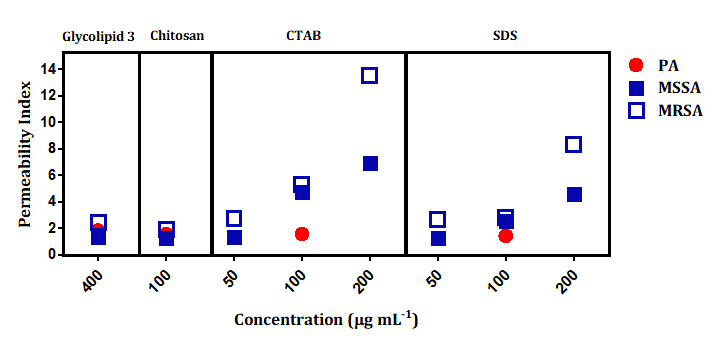


**Figure S6**. Influence of glycolipid **3** and other commonly known surfactants on the cell permeability of *Pseudomonas aeruginosa* (PA), Methicillin sensitive and resistant types of *Staphylococcus aureus* (MSSA and MRSA).


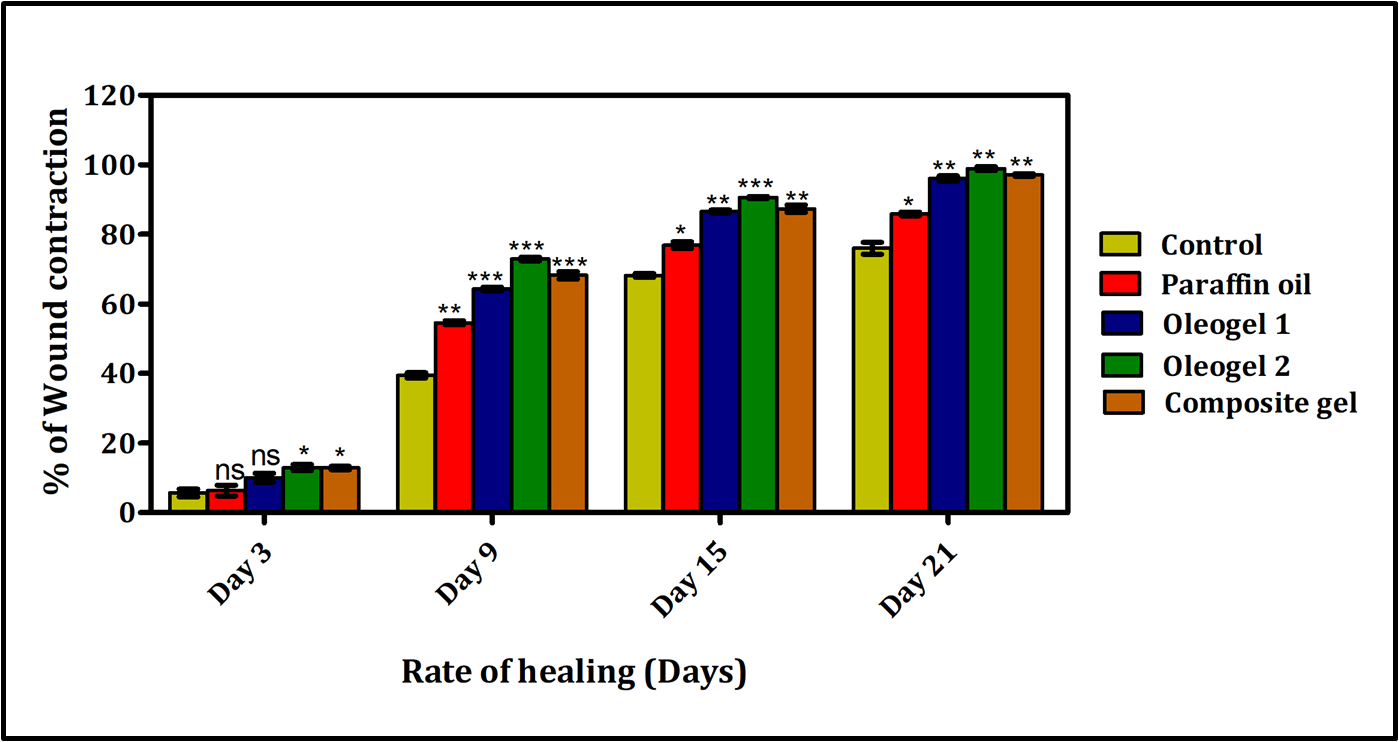


**Figure S7** Effect of oleogels and Composite gel on the percentage (%) of wound healing in diabetic induced rats with respective to days 3, 9, 15, and 21. Bars represent the percentage value of mean ± SEM of each group, n = 6. One way ANOVA followed by Dunnett’s test for multiple comparisons was performed to analyze the data. Significance between the control group with other treated groups is only depicted. (* *P* < 0.05, ** *P* < 0.01, *** *P* < 0.001).

**Figure S8.** Biochemical profile of granulation tissue obtained from the skin-excised wound of different experimental groups. Estimation of (a) hydroxyproline; (b) Hexosamine; (c) Ascorbic acid; (d) Lipid peroxidase. Bars represent values of mean ± SEM of each group, n = 6. One way ANOVA followed by Dunnett’s test for multiple comparisons was performed to analyze the data. Significance between the control group with other treated groups is only depicted. (* *P* < 0.05, ** *P* < 0.01, *** *P* < 0.001).


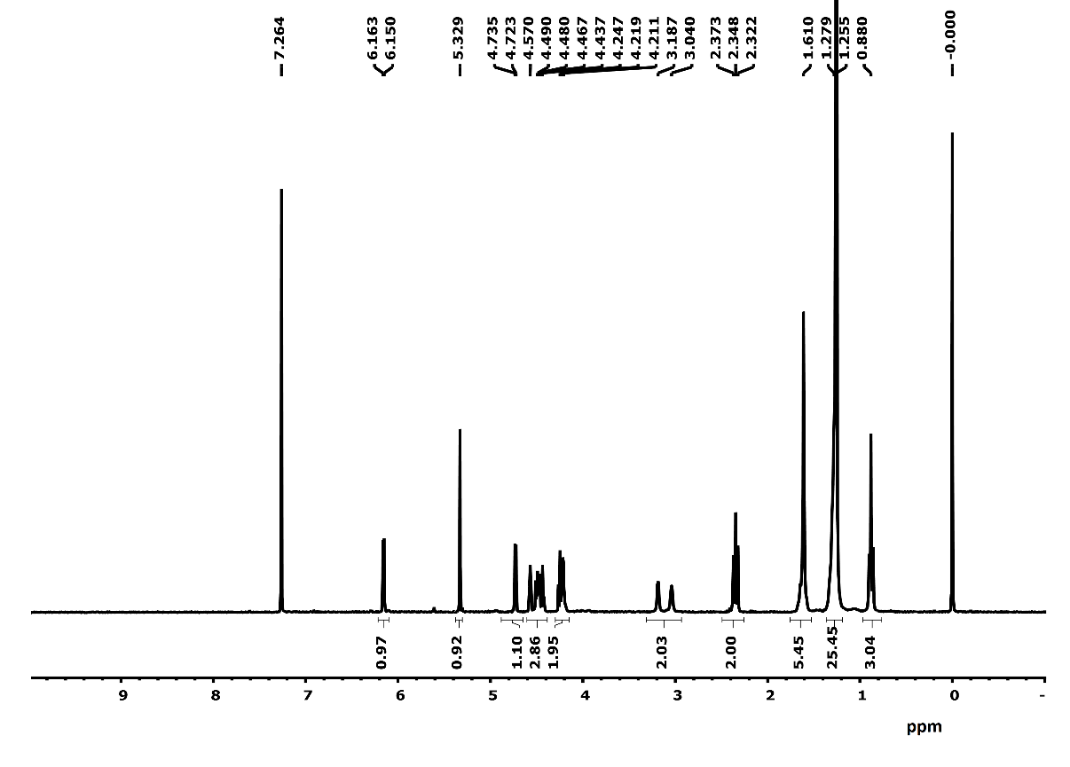


**Figure S9.** ^1^H NMR spectrum of glycolipid **3** in CDCl_3_


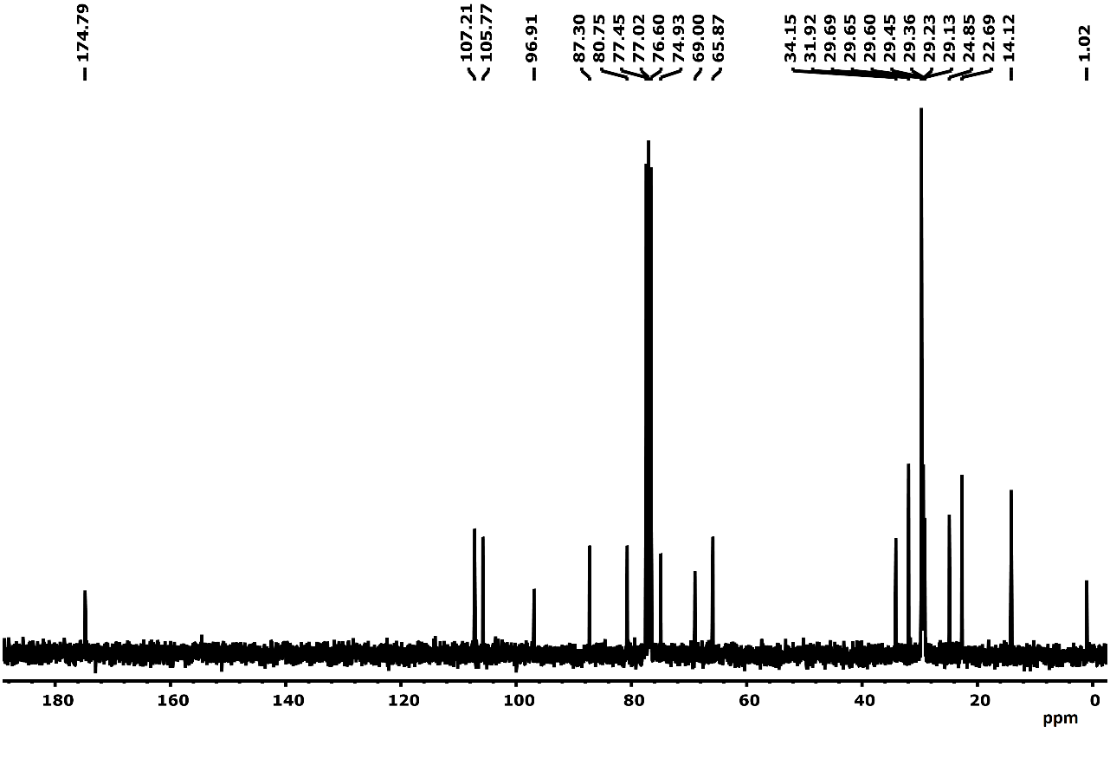


**Figure S10.** ^13^C NMR spectrum of glycolipid **3** in CDCl_3_

**Figure S11.** ESI-MS spectra of glycolipid **3** in CDCl_3_+MeOH^1^

REFERENCE

1. Siva Prasad, Y.; Saritha, B.; Tamizhanban, A.; Lalitha, K.; Kabilan, S.; Uma Maheswari, C.; Sridharan, V.; Nagarajan, S. Enzymatic Synthesis and Self-Assembly of Glycolipids: Robust Self-Healing and Wound Closure Performance of Assembled Soft Materials. *RSC Adv.* **2018**, *8*, 37136–37145.
2. Rybtke M. T., B. R. Borlee, K. Murakami, Y. Irie, M. Hentzer, T. E. Nielsen, M. Givskov, M. R. Parsek, and T. Tolker-Nielsen, “Fluorescence-based reporter for gauging cyclic Di-GMP levels in *Pseudomonas aeruginosa*,” *Appl. Environ. Microbiol.*, **2012,** vol. 78, no. 15, pp. 5060–5069.
3. Schwartbeck B., J. Birtel, J. Treffon, L. Langhanki, A. Mellmann, D. Kale, J. Kahl, N. Hirschhausen, C. Neumann, J. C. Lee, F. Götz, H. Rohde, H. Henke, P. Küster, G. Peters, and B. C. Kahl, “Dynamic *in vivo* mutations within the ica operon during persistence of *Staphylococcus aureus* in the airways of cystic fibrosis patients,” *PLoS Pathog.*, **2016,** vol. 12, no. 11.
4. Prasad Y. S., S. Miryala, K. Lalitha, K. Ranjitha, S. Barbhaiwala, V. Sridharan, C. U. Maheswari, C. S. Srinandan, and S. Nagarajan, “Disassembly of Bacterial Biofilms by the Self-Assembled Glycolipids Derived from Renewable Resources,” *ACS Appl. Mater. Interfaces,* **2017**, vol. 9, no. 46, pp. 40047–40058.
5. Niven G. W. and F. Mulholland, “Cell membrane integrity and lysis in *Lactococcus lactis*: The detection of a population of permeable cells in post-logarithmic phase cultures,” *J. Appl. Microbiol.*, **1998,** vol. 84, no. 1, pp. 90–96
